# Patient and Disease Characteristics Associated with Activation for Self-Management in Patients with Diabetes, Chronic Obstructive Pulmonary Disease, Chronic Heart Failure and Chronic Renal Disease: A Cross-Sectional Survey Study

**DOI:** 10.1371/journal.pone.0126400

**Published:** 2015-05-07

**Authors:** Irene Bos-Touwen, Marieke Schuurmans, Evelyn M. Monninkhof, Yvonne Korpershoek, Lotte Spruit-Bentvelzen, Inge Ertugrul-van der Graaf, Niek de Wit, Jaap Trappenburg

**Affiliations:** 1 Department of rehabilitation, nursing science & sports, University Medical Centre Utrecht, Utrecht, The Netherlands; 2 Julius Center for Health Sciences and Primary Care, University Medical Centre Utrecht, Utrecht, The Netherlands; Providence VA Medical Center and Brown University, UNITED STATES

## Abstract

A substantial proportion of chronic disease patients do not respond to self-management interventions, which suggests that one size interventions do not fit all, demanding more tailored interventions. To compose more individualized strategies, we aim to increase our understanding of characteristics associated with patient activation for self-management and to evaluate whether these are disease-transcending. A cross-sectional survey study was conducted in primary and secondary care in patients with type-2 Diabetes Mellitus (DM-II), Chronic Obstructive Pulmonary Disease (COPD), Chronic Heart Failure (CHF) and Chronic Renal Disease (CRD). Using multiple linear regression analysis, we analyzed associations between self-management activation (13-item Patient Activation Measure; PAM-13) and a wide range of socio-demographic, clinical, and psychosocial determinants. Furthermore, we assessed whether the associations between the determinants and the PAM were disease-transcending by testing whether disease was an effect modifier. In addition, we identified determinants associated with low activation for self-management using logistic regression analysis. We included 1154 patients (53% response rate); 422 DM-II patients, 290 COPD patients, 223 HF patients and 219 CRD patients. Mean age was 69.6±10.9. Multiple linear regression analysis revealed 9 explanatory determinants of activation for self-management: age, BMI, educational level, financial distress, physical health status, depression, illness perception, social support and underlying disease, explaining a variance of 16.3%. All associations, except for social support, were disease transcending. This study explored factors associated with varying levels of activation for self-management. These results are a first step in supporting clinicians and researchers to identify subpopulations of chronic disease patients less likely to be engaged in self-management. Increased scientific efforts are needed to explain the greater part of the factors that contribute to the complex nature of patient activation for self-management.

## Introduction

A rising problem in health care is the growing number of people with one or more chronic conditions [1]. Chronic conditions are associated with a higher mortality and a lower quality of life [2]. The growing number of chronic disease patients places a huge burden on our health care systems. A promising strategy to address the burden on patients and society is self-management. Self-management refers to ‘the individual’s ability to manage the symptoms, treatment, physical and psychosocial consequences and lifestyle changes inherent in living with a chronic condition’ [3].

In the last two decades, extensive scientific research on self-management has been performed and a wide range of self-management programs have been developed for various target populations. Results from meta-analyses show that self-management can improve quality of life, certain disease-specific outcomes and may reduce health care costs [4–6]. Although positive effects are seen in mean group outcomes, individual trials report that a substantial proportion of patients do not comply or respond to these interventions. This is demonstrated by the highly inconsistent outcomes in five clinical trials evaluating effectiveness of an almost identical self-management program in COPD patients ranging from highly successful to even harmful [7,8].

Little is known about characteristics distinguishing patients most likely to benefit from a self-management program. A first step in understanding variance in effect size is to identify factors associated with the degree to which patients are activated for self-management. Previous studies have identified several factors related to self-management behavior such as age [9], gender [9], education level [10], multi-morbidity [9], depression and anxiety [9,11,12], disease characteristics [10], illness perception [12], social support [13], disease duration, disease severity [9], low socio-economic-status [14] and health literacy [14]. These studies used highly varying methodologies and mainly focused on disease-specific parameters. We hypothesize that a substantial number of factors associated with levels of activation for self-management are disease-transcending instead of disease-specific, as many patients suffer from more than one chronic condition [15]. Previous studies analyzed only a limited number of factors simultaneously; ignoring a broader spectrum of disease and patient characteristics. Increased understanding of (disease-transcending) factors associated with activation for self-management is an essential first step towards tailored strategies.

This study aims to identify determinants of activation for self-management in patients with chronic diseases, i.e. patients with Chronic Obstructive Pulmonary Disease (COPD), Chronic Heart Failure (CHF), Diabetes Mellitus type II (DM-II) and Chronic Renal Disease (CRD). Furthermore, this study aims to identify variables associated with chronic disease patients at highest risk for low activation for self-management and to investigate whether these associations are disease-transcending.

## Materials and Methods

### Design and study population

A descriptive cross-sectional study was conducted to examine the association between determinants and outcome in four patient populations (COPD, CHF, DM-II and CRD). These diseases were selected as they are among the most prevalent in clinical practice and a substantial part of the disease progression and its burden can be delayed or partially prevented if patients engage in proactive management of their disease. Both determinants and outcome were assessed in a single questionnaire combined with chart review.

The study population consisted of adult patients living in the Netherlands with a clinical diagnosis of COPD, CHF, DM-II or CRD. Patients meeting the inclusion and exclusion criteria were recruited from six primary and four secondary care settings in different regions in the Netherlands. Inclusion criteria were adult patients with a clinical diagnosis of either COPD (age ≥40, post-bronchodilator FEV1/FVC ratio <70%), CHF (confirmed by clinical signs and symptoms and documented by a cardiologist), DM-II (fasting plasma glucose ≥7.0 mmol/l or 2h plasma glucose ≥11.1 mmol/l) or CRD (Glomerular Filtration Rate <60 mL/min/1.73m2). Exclusion criteria were being unable to speak, read, and write Dutch, conditions that could affect validity of the study (e.g. cognitive impairment), and having any kind of terminal illness (life expectancy < 3 months).

### Procedure/Ethics

Eligible patients were selected by chart review according to the inclusion and exclusion criteria. Patients received an invitation letter from their attending physician to participate in this study with the attached study information that explained determinants that were to be assessed as part of the study, an informed consent form and a questionnaire. Patients were asked to sign the informed consent form and to complete the questionnaire and return it to the research team. Patients were enrolled when both documents were received and subsequently chart review was performed. Patients who did not respond to the initial invitation received a reminder after three weeks. Permission for the study was granted by the medical ethical committee of the University Medical Center Utrecht.

### Outcome measure

The primary outcome was activation for self-management, which was assessed with the 13-item Patient Activation Measure (PAM-13). The PAM was considered the best generic measure, since it assesses self-reported knowledge, skills and confidence for self-management irrespective of the underlying chronic condition [16]. The PAM-13 has shown to be associated with a range of self-management behaviors (e.g. exercising and disease specific behaviors) [16,17]. A positive change in activation is related to a positive change in various self-management behaviors [18,19].

The PAM-13 is validated and translated into Dutch and has shown to be internally consistent (α = 0.88) with item-rest correlations varying from moderate to strong [20]. Answers are given on a five-point scale. The scores on each item are summed to give an overall raw score, this score is converted into a theoretical 0–100 point scale according to the PAM’s scoring table [21]. The PAM-13 makes a distinction among four activation levels associated with increasing self-management engagement. Based on cut-off points a person can be divided into level 1 (≤47.0 points), level 2 (47.1–55.1 points), level 3 (55.2–67 points) and level 4 (≥67.1 points) [17]. Level 1 includes the lowest activation scores corresponding to patients with low self-management engagement. These patients are not taking an active role in self-management and thus considered passive recipients of care [16].

### Determinants

Determinants of activation for self-management were assessed using various scales, surveys, sociodemographic and disease characteristics.

Health status was assessed using the Short Form-12 Health Survey (SF-12) [22], which is a shorter version of the Short Form-36 (SF-36) [23]. The SF-12 measures physical and mental health by means of two summary scores on a scale of 0–100. Higher scores on the SF-12 are related to a better health status. Results of the SF-12 and SF-36 showed almost complete overlap [24].

Anxiety and depression were measured with the Hospital Anxiety and Depression Scale (HADS) [25]. This is a 14-item self-reported screening instrument used as an indicator for the presence of anxiety or depression in general medical patients. The HADS includes two 7-item scales: one for anxiety and one for depression, both with a score range of 0–21. A higher rating indicates a higher state of anxiety or depression. A score ≥ 11 suggests the presence of an anxiety or depressive disorder. The HADS was validated in different groups of Dutch subjects [26].

Illness perception was measured with the Brief Illness Perception Questionnaire (B-IPQ) consisting of eight items that can be scored on a scale from 1–10. An overall score can be computed (scores range from 0–80) with a higher score reflecting a more threatening view of the disease. The B-IPQ was validated and translated into Dutch [27]. Face and content validity were found to be acceptable. Reproducibility showed moderate to good reliability [27].

Social support was assessed with the Multidimensional Scale of Perceived Social Support (MSPSS). This is a 12-item scale that was used to measure perceived social support [28]. The scale focuses on support from family, friends, and significant others. Items were scored on a 7-point Likert scale. Higher scores meant higher perceived support. The validity and reliability of the Dutch version MSPSS was confirmed in a group of Dutch cardiac patients and their partners [29].

Sociodemographic characteristics included age, gender, and ethnicity operationalized as Dutch versus any other nationality. Educational level, financial distress and a surrogate for income, namely receiving care allowance were measured to get an indication of socio-economic status. Educational level was divided into lower (primary school through vocational training), medium (secondary school or vocational training) and higher (college or university degree) education. Financial distress was defined as none, low or high distress. Receiving care allowance was operationalized as having a single annual income < €30,939 or a combined annual income < €42,438 [30].

Disease characteristics included severity and duration. Indicators of disease severity were operationalized by the stage of the disease (Table 1). Disease duration was divided in <2 years, 2–5 years or >5 years from diagnosis.

**Table 1:** Operationalization of disease severity.

Disease severity	DM-II	COPD	CHF	CRD
Mild	No medication	GOLD 1	NYHA 1	GFR 40–59 ml/min
Moderate	Only oral medication	GOLD 2 + 3	NYHA 2 + 3	GFR 15–39 ml/min
Severe	Use of insulin	GOLD 4	NYHA 4	GFR <15ml/min

DM-II = Diabetes mellitus type 2, COPD = Chronic obstructive pulmonary disease, CHF = Chronic Heart Failure, CRD = Chronic renal failure, GOLD = Global Initiative for chronic obstructive Lung Disease. NYHA = New York Heart Association. GFR = Glomerular Filtration Rate.

The impact of comorbidities was assessed using the Charlson Comorbidity Index [31]. This index is based on relative risks of mortality, in which ICD-10 conditions were assigned with values of 1, 2, 3, or 6 (all other conditions are given a score of 0) [32]. Comorbidities were extracted by chart review, the assigned values were then summed for each patient to create a total score.

### Data analysis

Analyses were performed using the SPSS Windows Version 20 [33]. Patient characteristics are presented as mean ± standard deviation for continuous variables and number and percentages for categorical variables. Patients were excluded when the PAM-13 (dependent variable) had more than seven missing items or when all 13 questions were answered identically [21]. Following analysis of missing values of all independent variables, multiple imputation was performed since imputation of missing values may reduce bias when the values are missing at random [34]. Ten imputated data-sets were created. Estimates for each data set were averaged to get a pooled estimate of the association. Statistical power was based on the rule of thumb of 10 events needed per variable [35].

Linear regression was performed to show the univariable determinant-outcome association and was not used as a selection method for candidate variables [36]. A multiple linear regression analysis with a stepwise backward method was performed to identify variables associated with activation for self-management. A p-value <0.20 was used for variable selection. This method was applied to each data set, resulting in 10 sets of selected variables. The final set comprised those variables that were selected in 50% or more of the 10 data sets [37]. The pooled results are presented. We used Fisher’s r to z transformation to calculate pooled R^2^ statistics as suggested by Harel [38]. The assumptions of linearity, homoscedasticity were checked and approved. A multiple correlation table was made of the studied determinants and the outcome and collinearity was considered when r > 0.8 (S1 Table). Since the assumption of normality was not completely met, generalized linear models were used with robust standard estimators in the linear regression analysis.

We assessed whether the associations between the determinants and activation were generic or disease specific by testing whether disease was an effect modifier. This was performed by comparing the R squared change of the final model with the same final model, but with the inclusion of an interaction term of one predictor variable with disease dummy variables. This procedure was repeated for all selected determinants. However, since patients could have more than one chronic disease under study and this could influence the results of this analysis we repeated this analysis in the subgroup of patients with only one studied disease.

To identify determinants associated with poor activation for self-management, PAM outcomes were dichotomized in patients in level 1 and patients in levels 2–4. Subsequently, a logistic regression analysis was performed and variables were identified using a similar methodology.

## Results

### Response and patient characteristics

**Fig 1:**
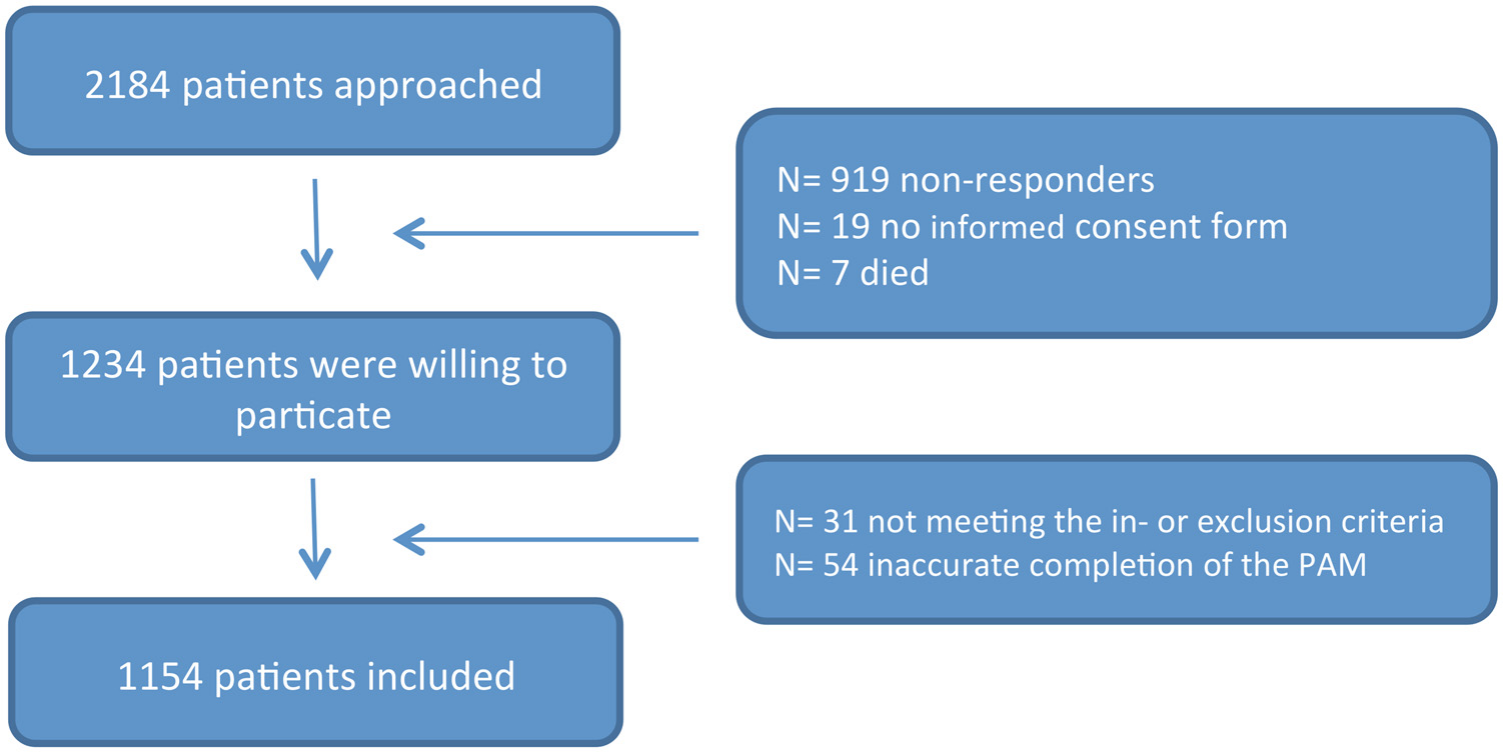
Flowchart of recruitment.

A total of 2184 eligible patients were approached to participate in this study of which 1239 patients were willing to participate (Fig 1). Of these patients, 31 patients were not eligible since they did not meet the inclusion and/or exclusion criteria. Another 54 patients produced invalid PAM-13 scores [21] and were, therefore, excluded. Data from 1154 patients (response rate 53%) were included in the final analysis. Of these 1154 patients, 422 (37%) were DM-II patients, 290 (25%) COPD patients, 223 (19%) CHF patients and 219 (19%) CRD patients. Of all participants, 60% were males and the age range was 28–92 years old with a mean age of 69.6±10.9. Almost 63% had more than 1 comorbid disease, and 30% of the patients had more than one of the four chronic diseases under study: 236 patients had 2, 97 patients had 3, and 13 patients had all four studied diseases. Patient characteristics and mean PAM-13 scores are described in Table 2. The percentage of total missing values was 2%; these missing values were distributed amongst 24% of our cases.

**Table 2:** Patient characteristics of the combined and separate chronic disease population(s).

*DM-II (n = 422)*		*COPD (n = 290)*	*CHF (n = 223)*	*CRD (n = 219)*	*Total (n = 1154)*
**Gender**					
Male	241 (57.1%)	184 (63.4%)	134 (60.1%)	135 (61.6%)	694 (60.1%)
Female	181 (42.9%)	105 (36.2%)	88 (39.5%)	84 (38.4%)	458 (39.7%)
**Age (years)**	68.0 ± 10.8	67.2 ± 10.3	73.9 ± 9.8	71.7 ± 11.5	69.6 ± 10.9
**Ethnicity**					
Dutch	402 (95.3%)	268 (92.4%)	212 (95.1%)	204 (93.2%)	1086 (94.1%)
Other	16 (3.8%)	19 (6.6%)	7 (3.1%)	12 (5.5%)	54 (4.7%)
**BMI (kg/m** ^**2**^ **)**	28.6 ± 4.3	26.6 ± 4.7	26.6 ± 4.3	27.8 ± 4.8	27.6 ± 4.6
**Living situation**					
Living alone	104 (24.6%)	72 (24.8%)	81 (36.3%)	66 (30.1%)	323 (28.0%)
Living not alone	313 (74.2%)	213 (73.4%)	137 (61.4%)	146 (66.7%)	809 (70.1%)
**Illness duration**					
≤2 years	80 (19.0%)	42 (14.5%)	43 (19.3%)	57 (26.0%)	222 (19.2%)
2–5 years	111 (26.3%)	69 (23.8%)	43 (19.3%)	76 (34.7%)	299 (25.9%)
>5 years	230 (54.5%)	135 (46.6%)	111 (49.8%)	59 (26.9%)	535 (46.4%)
**Illness severity**					
Mild	82 (19.4%)	93 (32.1%)	52 (23.3%)	54 (24.7%)	281 (24.4%)
Moderate	266 (63.0%)	173 (59.7%)	147 (65.9%)	133 (60.7%)	719 (62.3%)
Severe	43 (10.2%)	14 (4.8%)	15 (6.7%)	32 (14.6%)	104 (9.0%)
**Charlson comorbidity index**	2.1 ± 1.4	2.5 ± 1.5	3.8 ± 2.1	4.2 ± 1.7	2.9 ± 1.8
**Smoking**					
Never	126 (29.9%)	25 (8.6%)	62 (27.8%)	63 (28.8%)	276 (23.9%)
Former	236 (55.9%)	173 (59.7%)	135 (60.5%)	123 (56.2%)	667 (57.8%)
Current	54 (12.8%)	89 (30.7%)	24 (10.8%)	29 (13.2%)	196 (17.0%)
**Education**					
Low	201 (47.6%)	126 (43.4%)	103 (46.2%)	100 (45.7%)	530 (45.9%)
Medium	153 (36.3%)	111 (38.3%)	78 (35.0%)	87 (39.7%)	429 (37.2%)
High	58 (13.7%)	46 (15.9%)	37 (16.6%)	27 (12.3%)	168 (14.6%)
**Financial distress**					
None	211 (50.0%)	121 (41.7%)	105 (45.7%)	104 (47.5%)	538 (46.6%)
Low	157 (37.2%)	129 (44.5%)	100 (44.8%)	95 (43.4%)	481 (41.7%)
High	39 (9.2%)	33 (11.4%)	13 (5.8%)	12 (5.5%)	97 (8.4%)
Care Allowance*					
Received	219 (51.9%)	119 (41.0%)	135 (60.5%)	115 (52.5%)	588 (51.0%)
Not received	197 (46.7%)	159 (54.8%)	78 (35.0%)	101 (46.1%)	535 (46.4%)
**HADS**					
Depression	4.6 ± 3.6	5.6 ± 4.1	5.6 ± 4.0	5.1 ± 3.8	5.1 ± 3.9
Anxiety	4.3 ± 3.6	5.7 ± 4.3	5.4 ± 4.0	4.5 ± 3.6	4.9 ± 3.9
**Health status (SF-12)**					
Physical	60.9 ±25.6	45.6 ± 24.5	40.7 ±24.8	49.9 ±26.6	51.1 ± 26.3
Mental	69.8 ±23.0	61.7 ±23.4	59.7 ±24.8	66.2 ±23.1	65.2 ± 23.4
**Illness perception (B-IPQ)**	30.6 ±11.6	40.1 ±12.0	40.7 ± 11.2	37.8 ± 12.7	36.3 ± 12.7
**Social support (MSPSS)**	62.6 ± 15.0	60.6 ± 17.4	65.0 ± 15.0	64.0 ± 15.5	62.8 ± 15.8
**Comorbidity DM**		29 (10.1%)	67 (30.2%)	76 (34.7%)	
**Comorbidity COPD**	38 (9%)		42 (18.9%)	36 (16.4%)	
**Comorbidity CHF**	17 (4%)	14 (4.8%)		39 (17.8%)	
**Comorbidity CRD**	25 (5.9%)	9 (3.1%)	76 (34.2%)		
**Activation (PAM-13)**	55.3 ± 11.0	54.7 ± 10.4	53.6 ± 11.2	51.4 ± 10.0	54.1 ± 10.8

Data are presented as n, % or mean ± SD. DM-II = Diabetes Mellitus type II, COPD = Chronic obstructive Pulmonary Disease, CHF = Chronic Heart Failure, CRD = Chronic Renal Failure, BMI = Body Mass Index, HADS = Hospital Anxiety and Depression Scale, SF-12 = 12-item Short-form health survey, B-IPQ = Brief Illness Perception Questionnaire, MSPSS = Multidimensional Scale of Perceived Social Support, PAM-13 = 13-item Patient Activation Measure.

* Care allowance received by single people making an annual living < €30,939 or a combined annual living < € 42,438.

**Fig 2:**
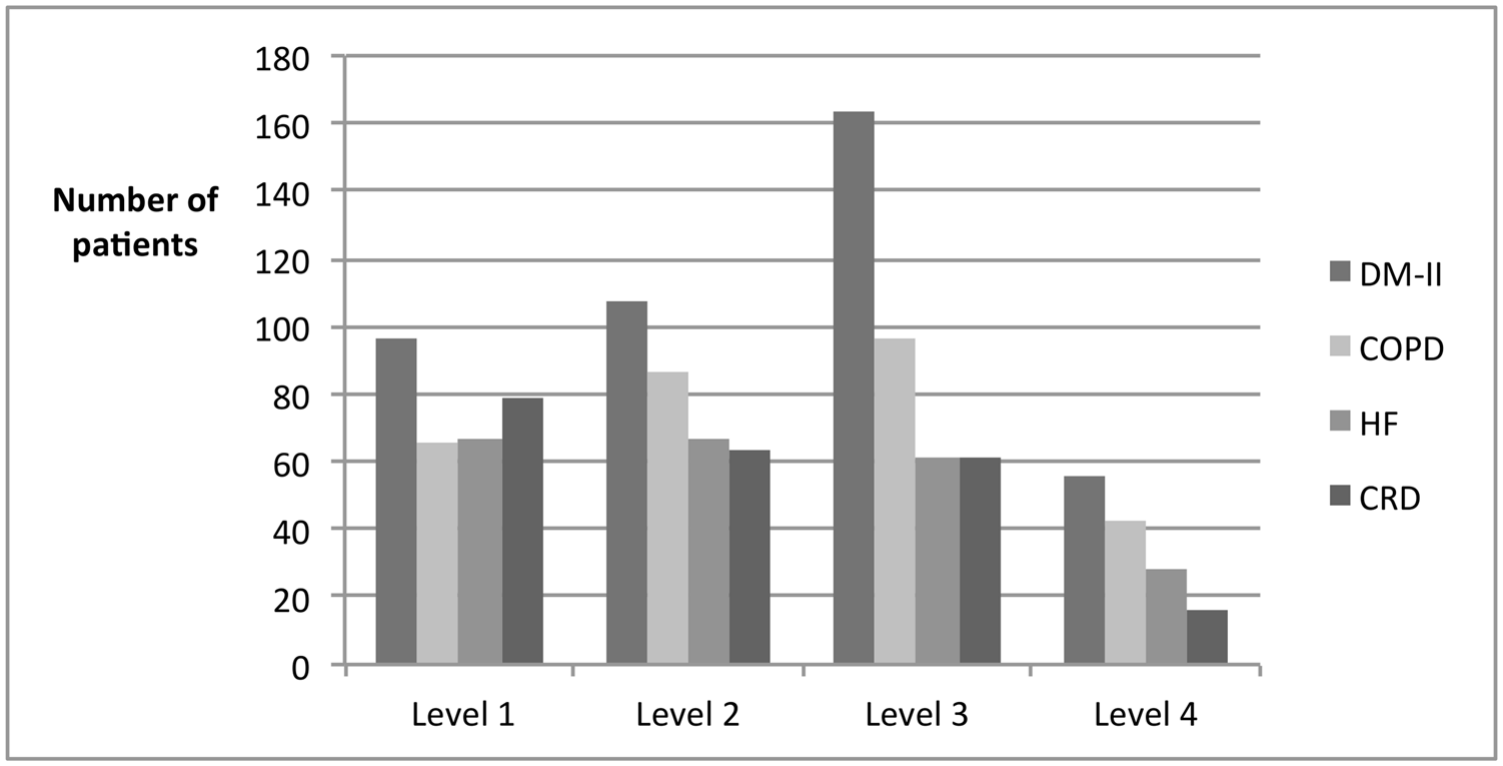
Distribution of PAM (patient activation measure) scores stratified by underlying chronic condition. DM-II = diabetes mellitus type 2, COPD = Chronic obstructive pulmonary disease, CHF = Chronic heart failure, CRD = Chronic renal disease.

Fig 2 shows the varying levels of self-management activation. Mean PAM-13 scores differed across the conditions: for DM-II the mean score was 55.3 ± 11.0, for COPD 54.7 ± 10.4, for CHF 53.6 ± 11.2 and for CRD 51.4 ± 10.0. For each condition, only a minority of patients scored PAM level four.

### Identifying variables associated with activation for self-management

Results of the univariable linear regression analyses are shown in Table 3 and of the multivariable linear regression analyses in Table 4. The explained variance (R^2^) of the full multivariable linear model was 0.17. Reducing the model by backward selection to a model with nine variables did not substantially change the explained variance (R^2^ 0.16). Variables associated with self-management activation in the final reduced multivariable model were age, BMI, education level, financial distress, physical health status, depression, illness perception, social support and chronic condition.

**Table 3:** Linear regression analyses—univariable associations of the determinants with activation for self-management.

	Univariable linear regression	
	Unstandardized coefficients (95% CI)	Standardized coefficients
**Age (years)**	-0.07 (-0.12;-0.01)	-0.07
**Gender (female vs male)**	-0.87 (-2.14; 0.40)	-0.04
**BMI (kg/m** ^**2**^ **)**	-0.33 (-0.46;-0.19)	-0.14
**Ethnicity (non-native vs native)**	-2.86 (-5.81; 0.09)	-0.06
**Level of education**		
Moderate vs low	0.17 (-1.18; 1.53)	0.01
High vs low	4.41 (2.56; 6.26)	0.15
**Living together vs alone**	1.37 (0.01;2.75)	0.06
**Smoking**		
Former vs never	0.35 (-1.17; 1.86)	0.02
Current vs never	-0.55 (-2.53; 1.43)	-0.02
**Financial distress**		
Low vs none	-3.46 (-4.77;-2.15)	-0.16
High vs none	-4.29 (-6.58;-2.00)	-0.11
**Receiving care allowance (y/n)**	-0.87 (-2.12; 0.39)	-0.04
**Charlson comorbidities index**	-0.83 (-1.16;-0.49)	-0.14
**Severity of disease**		
Moderate vs mild	-2.94 (-4.41;-1.48)	-0.13
Severe vs mild	-3.21 (-5.68;-0.74)	-0.09
**Duration of disease**		
2–5 yrs vs ≤2 yrs	1.04 (-0.81;2.88)	0.04
>5 yrs vs ≤2 yrs	0.17 (-1.48;1.83)	0.01
**Health status (SF-12)**		
Physical component	0.11 (0.09;0.13)	0.26
Mental component	0.11 (0.09;0.14)	0.24
**HADS (Hospital anxiety and depression scale)**		
Anxiety	-0.51 (-0.67;-0.36)	-0.19
Depression	-0.70 (-0.86;-0.55)	-0.25
**Illness perception**	-0.24 (-0.29;-0.19)	-0.28
**Social support**	0.12 (0.08;0.16)	0.18
**Chronic disease**		
COPD vs DM-II	-0.60 (-2.20;1.00)	-0.02
CHF vs DM-II	-1.73 (-3.47;0.01)	-0.06
CRD vs DM-II	-3.98 (-5.72;-2.23)	-0.14

CI = Confidence interval, BMI = Body Mass Index, DM-II = Diabetes Mellitus type II, COPD = Chronic Obstructive Pulmonary Disease, CHF = Chronic Heart Failure, CRD = Chronic Renal Failure.

**Table 4:** Linear regression analyses—multivariable associations of the determinants with activation for self-management.

	Final multiple linear regression model after reduction			Final multiple regression model +interaction term disease*social support		
	Unstandardized coefficients (95% CI)		Standardized coefficients	Unstandardized coefficients (95% CI)		Standardized coefficients
**Age (years)**	-0.04	(-0.10; 0.02)	0.04	-0.05	(-0.11; 0.01)	-0.05
**BMI (kg/m** ^**2**^ **)**	-0.24	(-0.37;-0.11)	-0.10	-0.24	(-0.38;-0.10)	-0.10
**Level of education**						
Moderate vs low	-0.57	(-1.81; 0.68)	-0.03	-0.40	(-0.17;0.90)	-0.02
High vs low	1.93	(0.10; 3.76)	0.06	2.02	(0.22;3.81)	0.07
**Financial distress**						
Low vs none	-1.72	(-2.98; -0.47)	-0.08	-1.67	(-2.95;-0.39)	-0.08
High vs none	-1.18	(-3.50; 1.15)	-0.03	-1.16	(-3.45;1.12)	-0.03
**Health status (SF-12)**						
Physical component	0.03	(-0.00;0.07)	0.08	0.03	(0.00;0.06)	0.08
**HADS (Hospital anxiety and depression scale)**						
Depression	-0.15	(-0.35;0.04)	-0.05	-0.14	(-0.34;0.06)	-0.05
**Illness perception**	-0.16	(-0.21;-0.10)	-0.19	-0.16	(-0.22;-0.10)	-0.19
**Social support**	0.09	(0.05;0.13)	0.13	0.15	(0.08;0.21)	0.21
**Chronic disease**						
COPD vs DM-II	1.35	(-0.23;2.94)	0.05	8.62	(2.73;14.51)	0.35
CHF vs DM-II	0.29	(-1.52; 2.10)	0.01	0.70	(-0.65;7.91)	0.03
CRD vs DM-II	-2.45	(-4.09;-0.81)	-0.09	3.31	(-3.73;10.35)	0.12
Social support*COPD vs DM-II				-0.12	(-0.21;-0.03)	-0.30
Social support*CHF vs DM-II				-0.01	(-0.12;0.10)	-0.02
Social support* CRD vs DM-II				-0.09	(-0.20;0.02)	-0.22
**Explained variance of the model**	R2 = 0.16, adjusted R2 = 0.15			R^2^ = 0.17, adjusted R^2^ = 0.16		

CI = Confidence Interval, BMI = Body Mass Index, DM-II = Diabetes Mellitus type II, COPD = Chronic Obstructive Pulmonary Disease, CHF = Chronic Heart Failure, CRD = Chronic Renal Failure.

Furthermore, we explored whether the associations between the determinants and activation were disease-transcending or disease-specific. All determinants in the final model, except social support, were associated with the PAM-13 irrespective of the underlying chronic condition. The relation between social support and activation differed per condition. In comparison to DM-II, there was a small negative interaction between social support and condition, particularly for COPD (model including COPD as interaction term: -0.12, 95%CI -0.21 to -0.03). This means that the association between COPD and activation is also dependent on social support. In the analysis in the subgroup of patients with only one studied condition (n = 806) we only found the same interaction for COPD and social support (S2 Table).

### Identifying patients at highest risk for poor activation for self-management

Table 5 shows results of the logistic regression analysis. Poor activation was associated with having a higher BMI, more financial distress, a higher comorbidity index score, a medium education level, a shorter disease duration, a more negative illness perception, living alone and being depressed.

**Table 5:** Multivariable logistic regression analyses—determinants associated with poor activation for self-management.

	Multivariable logistic regression
	OR (95% CI)
**BMI (kg/m** ^**2**^ **)**	1.05 (1.01; 1.08)
**Living alone vs together**	1.50 (1.10; 2.06)
**Financial distress**	
Low vs none	1.60 (1.17; 2.18)
High vs none	1.63 (0.98; 2.72)
**Charlson comorbidities index**	1.10 (1.00; 1.20)
**Education level**	
Moderate vs low	1.41 (1.03; 1.92)
High vs low	0.78 (0.47; 1.30)
**Duration of disease**	
2–5 yrs vs ≤2 yrs	0.67 (0.44; 1.02)
>5 yrs vs ≤2 yrs	0.66 (0.45; 0.96)
**Physical Health status (SF-12)**	0.99 (0.99; 1.00)
**HADS depression**	1.05 (1.01; 1.10)
**Illness perception**	1.03 (1.01; 1.04)
**Social support**	0.99 (0.98; 1.00)
**Chronic disease**	
COPD vs DM-II	0.67 (0.44; 1.01)
CHF vs DM-II	0.88 (0.56; 1.39)
CRD vs DM-II	1.27 (0.81; 2.00)
	Nagelkerke R^2^ = 0.20

CI = Confidence interval, OR = Odds ratio, CI = confidence interval, BMI = Body mass index, SF-12 = short form-12, HADS = Hospital anxiety and depression scale, DM-II = Diabetes Mellitus type II, COPD = Chronic Obstructive Pulmonary Disease, CHF = Chronic Heart Failure, CRD = Chronic Renal Failure.

## Discussion

This study aimed to identify variables associated with patient activation for self-management in combined chronic disease populations. In total, nine explanatory variables were found to be mildly associated with activation: age, BMI, education level, financial distress, physical health status, depression, illness perception, social support and disease. Although many variables were taken into account, the multiple linear regression model explained only 16% of the variance in self-management activation, which means that 84% remains unexplained. All variables in the model, except social support, were found to be disease-transcending.

Furthermore, variables associated with chronic disease patients at highest risk for poor activation for self-management were explored. These variables were BMI, living alone, education level, financial distress, comorbidity index score, disease duration, depression and illness perception. In this model most factors are the same, however living alone, comorbidity index score and disease duration are solely associated with poor activation.

The distribution in PAM levels showed that only a minority of the patients were in PAM level four and half of the patients were in level one and two, which suggests there is considerable room for improvement in activation for self-management in these patients. In this study, the mean scores on the PAM-13 are lower compared to another Dutch study by Rademaker et al, 54.1 vs 61.3 [20]. This might be explained by differences in patient population, such as a substantially lower age (59 vs 70 in the current study).

Previous studies have described several personal factors that influence (activation for) self-management. Most factors in this study are in line with other papers. Most studies investigated patients with one specific disease with fewer patient characteristics [10,14,39]. Previous studies using the PAM-13 showed that female patients, with a relatively younger age, a higher education level and a better self-reported health had higher PAM-13 scores [16]. The same associations were described by Rademaker et al. in a Dutch sample, except that male gender, instead of female, was associated with a higher PAM-13 score [20]. In this study, gender was not associated with PAM-13 scores; the other associations were similar. In line with our study, Hibbard et al. found that having depressive symptoms is related to a lower PAM-13 score [16].

This study aimed to identify variables independently associated with activation for self-management. Although we studied a large number of variables, only 16% of the variance could be explained. This is in line with the study of Rockwell et al. reporting a low explained variance of 10.3% [10]. Rockwell et al. tested a model of seven patient characteristics in CHF patients and found that only two characteristics were positively associated with self-management of heart failure; a higher education level and increased disease severity. In this study, disease severity did not remain in the model as an explanatory variable. Another CHF study explained 38% of the variance of self-care management [9] with the following four variables: gender, moderate-to-severe comorbidity, depression, and self-care confidence. They found that male gender and moderate-to-severe comorbidity index was associated with higher self-care scores. In our study, patients with a higher comorbidity index had lower levels of activation for self-management. The differences in identified determinants and explained variance could possibly be explained by the large heterogeneity in outcome measures and the chosen selection of variables.

With a composition of 19 disease- and patient specific variables in the model, we expected to explain a greater amount of the variance in the PAM-13 score. However, linear associations of variables and PAM-13 were low. It can be concluded that engagement in self-management is a complex and perhaps even diffuse behavior not easy to grasp with straightforward disease- and patient parameters. An important finding is that the associations found were disease-transcending, which means that their impact on varying levels of activation for self-management is not modified by the underlying chronic condition. The main question remaining is which factors can explain the remaining 84% in our model. According to Bandura’s social cognitive theory [40], individuals are most likely to engage in a health behavior if they are confident in their ability to perform the behavior, in other words self-efficacy. Self-efficacy was not included in this analysis because self-efficacy items are included in the PAM-13. The Transtheoretical Model of behavioral change, as described by Prochaska et al., was used to develop the PAM-13 [41]. This model emphasizes motivation and readiness to move forward on the different stages of change. Motivation is a frequently mentioned factor facilitating self-management engagement in qualitative research [42,43]. However, we did not measure motivation as a separate variable. Motivation and self-efficacy could have contributed to explain the remaining variance. Other factors that we did not incorporate into our model and that could have contributed are health literacy [20] and cognitive impairment [44], since they have shown to be associated with activation for self-management.

A strength of this study is the large sample size and the relatively high response rate. Furthermore, the sample consisted of four different chronic disease populations, and we included patients from primary and secondary care settings, thus creating a wide range in disease severity.

A limitation is the use of self-reported questionnaires which might have introduced misclassification due to patients’ tendency to provide social desirable answers and may, therefore, have led to underestimation of effects. Due to the cross-sectional design, no conclusions can be drawn on the cause and effect of the associations. Furthermore, as a result of the survey design, we could not include all potential important determinants. Patients with cognitive impairment were excluded because these impairments may lead to less valid answers on the questionnaire. Furthermore, few non-native patients were included, which is probably due to the language barrier to fill out a questionnaire.

By trying to find generic variables across four chronic diseases a generic questionnaire, the PAM-13, was chosen as a measure for activation for self-management. We considered this tool to be the best available method to evaluate the complexity of activation for self-management in different target populations.

Future longitudinal studies are needed to investigate whether the identified variables can be used to identify subgroups of patients with barriers to engage in self-management. Future studies should evaluate the importance of other factors, such as cognitive impairment, health literacy, motivation and self-efficacy with regard to self-management activation. Additionally, scientific efforts are needed to investigate causal pathways between these determinants and activation. This knowledge might help healthcare professionals to identify patients at risk of inadequate self-management behaviors, which is essential for the development of more individually targeted and tailored interventions.

## Conclusions

This study increases the understanding about the distribution of activation for self-management in a large population of chronic disease patients. Irrespective of the underlying condition, a substantial part of patients register low PAM scores, indicating poor levels of activation. The results show that age, BMI, education level, financial distress, physical health status, depression, illness perception, social support and disease are associated with activation for self-management and that BMI, living alone, education level, financial distress, comorbidity index score, disease duration, depression and illness perception are associated with poor self-management engagement. As expected, most variables in the model were disease-transcending, except social support. This knowledge is a first step in helping health care providers to identify subpopulations of chronic disease patients that are less likely to be engaged in self-management activities which is essential to move towards targeting and tailoring of self-management interventions. However, we are still a long way from explaining the greater part of factors that contribute to the complex nature of self-management behavior.

## Supporting Information

S1 Table Multiple correlation table. HADS = Hospital Anxiety and Depression Scale, COPD = Chronic Obstructive Pulmonary disease. * p-value <0.01.
(DOCX)

S2 Table Linear regression analyses—multivariable associations of the determinants with activation for self-management in patients with no comorbid study diseases. CI = Confidence interval, BMI = Body Mass Index, DM-II = Diabetes Mellitus type II, COPD = Chronic Obstructive Pulmonary Disease, CHF = Chronic Heart Failure, CRD = Chronic Renal Failure.
(DOCX)
