# Supplementary material for: Patient and Disease Characteristics Associated with Activation for Self-Management in Patients with Diabetes, Chronic Obstructive Pulmonary Disease, Chronic Heart Failure and Chronic Renal Disease: A Cross-Sectional Survey Study
Source: PLoS One. 2015 May 7;10(5):e0126400. doi: 10.1371/journal.pone.0126400 (PMC4423990; doi:10.1371/journal.pone.0126400)
Supplement: S1 Table — HADS = Hospital Anxiety and Depression Scale, COPD = Chronic Obstructive Pulmonary disease. * p-value <0.01. (DOCX) [file pone.0126400.s001.docx]

**S1 Table.** Multiple correlation table

|  | Correlations | | | | | | | | | | | | | | | | | | | | | | |
| --- | --- | --- | --- | --- | --- | --- | --- | --- | --- | --- | --- | --- | --- | --- | --- | --- | --- | --- | --- | --- | --- | --- | --- |
|  | 1 | 2 | 3 | 4 | 5 | 6 | 7 | 8 | 9 | 10 | 11 | 12 | 13 | 14 | 15 | 16 | 17 | 18 | 19 | 20 | 21 | 22 | 23 |
| 1 Activation | 1 |  |  |  |  |  |  |  |  |  |  |  |  |  |  |  |  |  |  |  |  |  |  |
| 2 Gender | -0.04 | 1 |  |  |  |  |  |  |  |  |  |  |  |  |  |  |  |  |  |  |  |  |  |
| 3 Age | -0.07 | 0.00 | 1 |  |  |  |  |  |  |  |  |  |  |  |  |  |  |  |  |  |  |  |  |
| 4 Ethnicity | -0.06 | -0.05 | -0.11* | 1 |  |  |  |  |  |  |  |  |  |  |  |  |  |  |  |  |  |  |  |
| 5 Body mass index | -0.14* | 0.04 | -0.13* | 0.03 | 1 |  |  |  |  |  |  |  |  |  |  |  |  |  |  |  |  |  |  |
| 6 Living situation | 0.06 | -0.18* | -0.20* | 0.04 | 0.01 | 1 |  |  |  |  |  |  |  |  |  |  |  |  |  |  |  |  |  |
| 7 Illness duration | -0.00 | 0.00 | 0.13* | -0.02 | 0.04 | -0.06 | 1 |  |  |  |  |  |  |  |  |  |  |  |  |  |  |  |  |
| 8 Illness severity | -0.11* | -0.03 | 0.06 | 0.08* | 0.02 | -0.03 | 0.23* | 1 |  |  |  |  |  |  |  |  |  |  |  |  |  |  |  |
| 9 Charlson comorbidity index | - 0.14* | -0.08* | 0.34* | -0.04 | 0.01 | -0.14* | 0.07 | 0.11* | 1 |  |  |  |  |  |  |  |  |  |  |  |  |  |  |
| 10 Smoking | -0.01 | -0.17* | -0.13* | -0.01 | -0.08* | 0.02 | -0.08 | 0.00 | 0.02 | 1 |  |  |  |  |  |  |  |  |  |  |  |  |  |
| 11 Education | 0.11* | -0.16* | -0.11* | 0.00 | -0.12* | 0.04 | 0.00 | -0.07 | -0.06 | -0.03 | 1 |  |  |  |  |  |  |  |  |  |  |  |  |
| 12 Financial distress | -0.16* | 0.06 | -0.11* | 0.18* | 0.17* | -0.05 | 0.03 | 0.06 | 0.06 | 0.09 | -0.21 | 1 |  |  |  |  |  |  |  |  |  |  |  |
| 13 Care allowance | -0.04 | 0.04 | 0.04 | 0.03 | 0.05 | 0.14 | 0.04 | 0.03 | 0.08 | -0.04 | 0.00 | 0.05 | 1 |  |  |  |  |  |  |  |  |  |  |
| 14 HADS Depression | -0.25* | 0.07 | 0.11* | 0.18* | 0.07 | -0.06 | 0.04 | 0.15* | 0.20* | 0.00 | -0.19* | 0.27* | 0.08* | 1 |  |  |  |  |  |  |  |  |  |
| 15 HADS anxiety | -0.18* | 0.15* | 0.03 | 0.17* | 0.04 | -0.11* | 0.01 | 0.12* | 0.14* | 0.03 | -0.12* | 0.25* | 0.05 | 0.66* | 1 |  |  |  |  |  |  |  |  |
| 16 Physical health status | 0.26* | -0.15* | -0.22* | -0.10* | -0.12* | 0.15* | -0.08 | -0.22* | -0.33* | 0.01 | 0.12* | -0.24* | -0.07 | -0.56* | -0.48* | 1 |  |  |  |  |  |  |  |
| 17 Mental health status | 0.24* | -0.23* | -0.11* | -0.14* | -0.08* | 0.14* | -0.06 | -0.20* | -0.22* | 0.02 | 0.14* | -0.26* | -0.08 | -0.71 | -0.67* | 0.75* | 1 |  |  |  |  |  |  |
| 18 Illness perception | -0.28* | 0.03 | 0.00 | 0.14* | 0.01 | -0.04 | 0.06 | 0.22* | 0.16* | 0.03 | -0.10* | 0.18* | 0.03 | 0.49* | 0.43* | -0.53* | -0.51* | 1 |  |  |  |  |  |
| 19 Social support | 0.18 | -0.05 | -0.01 | -0.07 | -0.03 | 0.19* | 0.01 | 0.04 | 0.03 | -0.04 | -0.01 | -0.10* | 0.04 | -0.25* | -0.20* | 0.13* | 0.21* | -0.12* | 1 |  |  |  |  |
| 20 Diabetes mellitus type 2 | 0.00 | -0.00 | 0.03 | 0.01 | 0.26* | -0.02 | 0.09* | 0.12* | 0.05 | -0.12* | -0.05 | 0.02 | 0.12 | -0.05* | -0.09* | 0.13* | 0.08* | -0.26* | -0.03 | 1 |  |  |  |
| 21 COPD | 0.00 | -0.08 | -0.04 | 0.03 | -0.09* | -0.00 | 0.05 | -0.06 | 0.08* | 0.31* | -0.01 | 0.09 | -0.06 | 0.10* | 0.13* | -0.20* | -0.13* | 0.20* | -0.06 | -0.39* | 1 |  |  |
| 22 Heart failure | -0.06 | -0.01 | 0.26* | -0.03 | -0.08 | -0.09 | 0.02 | 0.04 | 0.38* | -0.09* | -0.01 | 0.02 | 0.12* | 0.10* | 0.07 | -0.24* | -0.13* | 0.16* | 0.10* | -0.19* | -0.14* | 1 |  |
| 23 Chronic renal disease | -0.14* | -0.03 | 0.22* | -0.01 | 0.01 | -0.08 | -0.08* | 0.08* | 0.57* | -0.08 | -0.02 | -0.03 | 0.05 | 0.05 | -0.00 | -0.13* | -0.05 | 0.12* | 0.07 | -0.12* | -0.19* | 0.17* | 1 |
