# Supplementary material for: Patient and Disease Characteristics Associated with Activation for Self-Management in Patients with Diabetes, Chronic Obstructive Pulmonary Disease, Chronic Heart Failure and Chronic Renal Disease: A Cross-Sectional Survey Study
Source: PLoS One. 2015 May 7;10(5):e0126400. doi: 10.1371/journal.pone.0126400 (PMC4423990; doi:10.1371/journal.pone.0126400)
Supplement: S2 Table — CI = Confidence interval, BMI = Body Mass Index, DM-II = Diabetes Mellitus type II, COPD = Chronic Obstructive Pulmonary Disease, CHF = Chronic Heart Failure, CRD = Chronic Renal Failure. (DOCX) [file pone.0126400.s002.docx]

|  | **Final multiple linear regression model after reduction** | | **Final multiple**  **regression model**  **+interaction term**  **disease*Social support** | |
| --- | --- | --- | --- | --- |
| **N=806** | **Unstandardized coefficients (95% CI)** | **Standardized coefficients** | **Unstandardized coefficients (95% CI)** | **Standardized coefficients** |
| **BMI (kg/m^2^)** | -0.14 (-0.31;0.03) | 0.06 | -0.13 (-0.30;0.04) | -0.05 |
| **Level of education** |  |  |  |  |
| Moderate vs low | 0.40 (-1.14; 1.94) | 0.02 | -0.63 (-0.91;2.17) | 0.03 |
| High vs low | 3.30 (1.22; 5.37) | 0.11 | 3.45 (1.38; 5.53) | 0.12 |
| **Financial distress** |  |  |  |  |
| Low vs none | -1.40 (-2.92; 0.12) | -0.07 | -1.33 (-2.85;0.19) | -0.06 |
| High vs none | -1.16 (-3907; 1.56) | -0.03 | -0.95 (-3.68;1.77) | -0.03 |
| **Health status (SF-12)** |  |  |  |  |
| Physical component | 0.05 (0.01;0.08) | 0.11 | 0.04 (0.11;0.08) | 0.11 |
| **Illness perception** | -0.15 (-0.22;-0.08) | -0.18 | -0.15 (-0.22;-0.08) | -0.18 |
| **Social support** | 0.09 (0.05;0.14) | 0.14 | 0.16 (0.09;0.24) | 0.24 |
| **Chronic disease** |  |  |  |  |
| COPD vs DM-II | 1.79 (-0.14;3.59) | 0.08 | 10.47 (4.03;16.91) | 0.45 |
| CHF vs DM-II | 0.97 (-1.37; 3.32) | 0.03 | 3.19 (-6.76; 13.13) | 0.10 |
| CRD vs DM-II | -1.72 (-3.93;0.94) | -0.06 | 3.36 (-6.20;12.93) | 0.11 |
| **Social support*COPD vs DM-II** |  |  | -0.14 (-0.24;-0.41) | -0.39 |
| **Social support*CHF vs DM-II** |  |  | -0.04 (-0.19;0.11) | -0.08 |
| **Social support* CRD vs DM-II** |  |  | -0.08 (-0.23; 0.06) | -0.18 |
| Explained variance of the model | R^2^= 0.14,  adjusted R^2^=0.13 |  | R^2^= 0.15,  adjusted R^2^=0.13 | |
